# Concurrent Imatinib and Radiation Therapy for Unresectable and Symptomatic Desmoid Tumors

**DOI:** 10.1155/2017/2316839

**Published:** 2017-07-05

**Authors:** Everett J. Moding, Lynn Million, Raffi Avedian, Pejman Ghanouni, Christian Kunder, Kristen N. Ganjoo

**Affiliations:** ^1^Department of Radiation Oncology, Stanford University School of Medicine, Stanford, CA, USA; ^2^Department of Orthopaedic Surgery, Stanford University School of Medicine, Stanford, CA, USA; ^3^Department of Radiology, Stanford University School of Medicine, Stanford, CA, USA; ^4^Department of Pathology, Stanford University School of Medicine, Stanford, CA, USA; ^5^Department of Medicine, Division of Oncology, Stanford University School of Medicine, Stanford, CA, USA

## Abstract

Desmoid tumors are locally aggressive fibroproliferative neoplasms that can lead to pain and dysfunction due to compression of nerves and surrounding structures. Desmoid tumors often progress through medical therapy, and there is frequently a delay of multiple months before radiation can provide symptomatic relief. To achieve more rapid symptomatic relief and tumor regression for unresectable desmoid tumors causing significant morbidity such as brachial plexus impingement with loss of extremity function, we have selectively utilized a combination of imatinib and radiation therapy. Here, we retrospectively review four patients treated with concurrent imatinib and radiation therapy. The treatment was typically tolerated with minimal toxicity though one patient developed avascular necrosis of the irradiated humeral head possibly related to the combined treatment. All the patients treated have had a partial response or stable disease on imaging. Improvement of symptoms was observed in all the treated patients with a median time to relief of 2.5 months after starting radiation therapy. Concurrent radiation and imatinib may represent a viable treatment option for unresectable and symptomatic desmoid tumors where rapid relief is needed to prevent permanent loss of function.

## 1. Introduction

Desmoid tumors (also known as desmoid-type fibromatosis or aggressive fibromatosis) can arise anywhere in the body and are locally aggressive, leading to significant morbidity due to their large size and pain caused by compression and infiltration of surrounding nerves and normal structures [1]. Management of desmoid tumors is individualized based on age at diagnosis, tumor location, underlying genetics, and symptoms at presentation [2]. For progressive or symptomatic tumors, multiple treatment modalities including systemic therapies, surgical resection, and radiation therapy are available, but all are prone to local recurrence and associated treatment with treatment related toxicity [3].

Our current treatment approach for adults with extra-abdominal desmoid tumors that are symptomatic or progressive is to choose therapy that minimizes normal tissue injury while maximally preserving organ/limb function. For example, we have employed noninvasive treatment modalities such as MR-guided high-intensity focused ultrasound to avoid surgery for extremity desmoid tumors [4, 5]. We consider surgery and/or radiation therapy for desmoid tumors that are symptomatic and not responsive to medical therapy. However, we have recently identified a subset of patients with desmoid tumors of the trunk/shoulder girdle that cause significant pain due to their deep involvement of the paraspinal muscles or the neurovascular bundle within the axilla. Due to location and infiltration of normal structures, surgical resection would lead to unacceptable morbidity.

Imatinib is a tyrosine kinase inhibitor that has been utilized as a monotherapy for desmoid tumors based on positive immunohistochemical staining and qualitative real-time PCR suggesting overexpression of c-KIT, platelet-derived growth factor receptor PDGFR-*α*, and PDGFR-*β* [6]. More recently, three phase II trials of imatinib monotherapy have demonstrated that imatinib is effective at delaying progression of some desmoid tumors [7–9], although there was no consistent correlation between expression of known imatinib targets and response to therapy. Several studies have shown that radiation therapy leads to excellent rates of local control for desmoid tumors both alone and in combination with surgery [10]. Although studies have not reported on time until symptom relief after radiation therapy, it can often take several months before desmoid tumors respond to radiation therapy, leading to prolonged symptoms and morbidity. A retrospective review demonstrated that radiation doses greater than 56 Gy were associated with greater complications without increasing local control [11], and NCCN guidelines currently recommend treating unresectable desmoid tumors with 54–58 Gy.

Preclinical studies have suggested that imatinib may potentiate the effects of radiation therapy in other tumor types. This effect has been attributed to reduced cell proliferation [12, 13], endothelial cell death [14], inhibition of PDGFR [12, 15], and inhibition of homologous recombination [16]. Based on these preclinical studies, we hypothesized that imatinib may provide rapid growth arrest of the desmoid tumor while it takes months for tumor shrinkage from radiation therapy leading to more rapid symptom relief for patients with unresectable and symptomatic desmoid tumors. Furthermore, we anticipated a radio-sensitization effect when combined with imatinib allowing slightly lower doses of radiation to limit normal tissue injury of adjacent critical structures such as the spinal cord. Here, we present a retrospective series of 4 such patients that we have treated with concurrent imatinib and radiation therapy.

## 2. Materials and Methods

### 2.1. Study Design

This study was approved by the Stanford Institutional Review Board under protocol 39030. We utilized the Stanford Translational Research Integrated Database Environment (STRIDE) [17] to help retrospectively identify patients seen in consultation at the Stanford University Cancer Center with unresectable desmoid tumors who were treated with concurrent imatinib and radiation therapy since 2014. The sample size was not predetermined. Patients were excluded if there was insufficient follow-up to assess the response to treatment and/or development of side effects.

### 2.2. Treatment

Patients were started on imatinib two to seven weeks prior to initiating radiation therapy. Imatinib was initiated at 300 or 400 mg daily, and the dose was titrated down to 200 mg daily in two patients due to nausea. Imatinib was continued indefinitely or until side effects became intolerable, but all patients received at least 6 months of imatinib treatment. Three of the patients received radiation therapy at Stanford Health Care, and one patient was treated at an outside facility. External beam radiation therapy to 50–54 Gy was delivered in 1.8–2 Gy daily fractions using either a 3D conformal or intensity modulated radiation therapy technique. Care was taken to limit radiation dose to surrounding normal structures including adjacent joints when possible.

### 2.3. Assessment of Response

Radiation and imatinib toxicity were assessed based on clinical notes according to the Common Terminology Criteria for Adverse Events (CTCAE), Version 4.0. Time to first improvement of symptoms was calculated from the first day of radiation treatment based on available clinical notes. The changes in tumor volume and largest diameter after treatment were measured by the study investigators and compared with the most recent CT or MRI prior to completing radiation therapy. Treatment response was determined based on the revised Response Evaluation Criteria In Solid Tumors (RECIST) [18]. Follow-up time was calculated from the completion of radiation therapy to the most recent MRI or CT imaging.

## 3. Results

### 3.1. Patient Characteristics

A total of 11 patients were identified who had undergone treatment with concurrent imatinib and radiation. Two were excluded due to insufficient follow-up, four patients had undergone prior resection or treatment, and one patient's tumor was not a desmoid tumor on review of outside pathology. Two women and two men treated with concurrent imatinib and radiation therapy were analyzed (Table 1). The median age of the patients was 57.5 years old (range 47–70). Two patients had desmoid tumors located along the posterior thorax invading paraspinal muscles and two patients had axillary primary sites with tumor infiltrating the brachial neurovascular bundle. None of the patients had received previous treatment. All the tumors were associated with significant pain. One patient had associated neurologic impairment including ipsilateral upper extremity numbness and weakness. The patients were symptomatic for a median duration of 6 months prior to initiating radiation therapy (range 2 to 51 months). Surgical consults confirmed that surgical resection would have led to unacceptable morbidity due to proximity to critical structures.

### 3.2. Treatment Toxicity

The combined treatment was generally well tolerated (Table 1). The most common side effects attributable to radiation were fatigue and dermatitis, and imatinib was associated with nausea and fatigue. One patient with a left axillary desmoid tumor developed ipsilateral breast edema 4 months after radiation therapy that resolved with physical therapy. Another patient with an axillary desmoid tumor was noted to have radiographic features of avascular necrosis and adhesive capsulitis of the humeral head on MRI 4 months after radiation therapy. Imatinib was stopped, and the patient has been managed medically.

### 3.3. Response to Treatment

**Figure 1:**
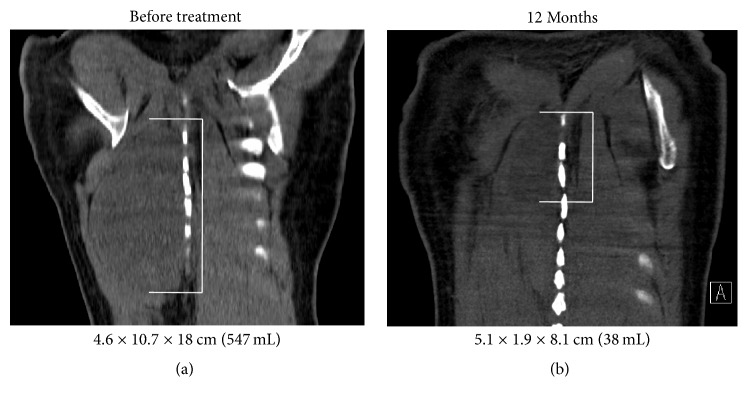
Imaging of Posterior Thorax Desmoid Treatment Response. Coronal CT of a right paraspinal desmoid tumor before (a) and 12 months after (b) completing radiation therapy to 50 Gy in 25 fractions with concurrent imatinib demonstrating a significant reduction in tumor volume. The tumor is delineated with white brackets.

**Figure 2:**
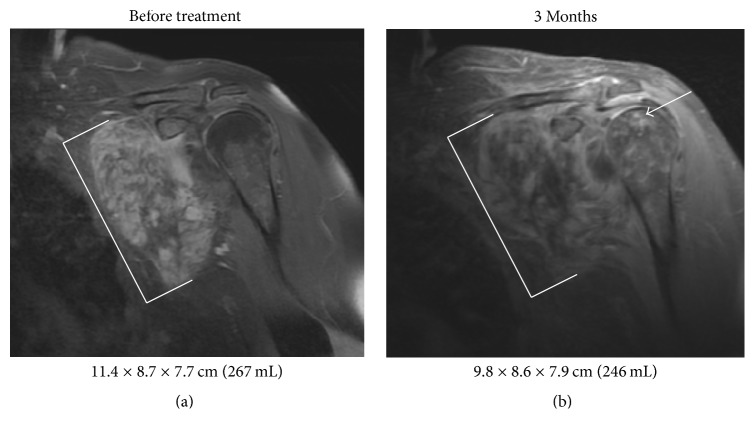
Imaging of Axillary Desmoid Treatment Response. Coronal T1 postcontrast MRI of a left axillary desmoid tumor before (a) and 3 months after (b) completing radiation therapy to 50.4 Gy in 28 fractions with concurrent imatinib demonstrating decreased enhancement with minimal change in tumor size. The tumor is delineated with white brackets. Imaging changes noted in the humeral head compatible with avascular necrosis are indicated with the white arrow.

All the patients had improvement of their symptoms with a median time to relief of 2.5 months after starting radiation therapy (range 0–11 months). At a median follow-up of 8 months since completing radiation (range 3–18 months), all the patients have stable disease or a partial response by RECIST criteria with a median reduction in tumor volume of 49% (range 8–93%). The patient with the largest change in tumor volume experienced improvement in his symptoms 7 days after starting radiation therapy to his thoracic mass (Figure 1). One patient with stable disease in the left axilla had good symptom relief with decreased contrast enhancement of her desmoid tumor on MRI (Figure 2).

## 4. Discussion

In the four patients described here, concurrent imatinib and radiation therapy was effective at relieving pain and improving neurologic symptoms attributed to large unresectable desmoid tumors. None of the patients developed disease progression after treatment, and one patient had a dramatic decrease in tumor volume.

Previous prospective studies have demonstrated one-year local control rates of 36.8% to 67% with imatinib monotherapy for desmoid tumors [7–9]. A prospective phase II study of radiation therapy with 56 Gy in 28 fractions alone for inoperable desmoid tumors found a three-year local control rate of 81.5% with best overall response being complete response in 13.6%, partial response in 36.4%, stable disease in 40.9%, and progressive disease in 6.8% of patients [19]. Given the delay in tumor response with radiation therapy alone, imatinib may prevent further progression of tumors until radiation can take effect. For example, one study showed a median time to first regression of 4 months after radiation therapy with one patient taking 26 months until first regression [11]. Given the small number of patients in this retrospective series, it is difficult to conclusively compare concurrent imatinib and radiation therapy with radiation or imatinib monotherapy, but our results compare favorably with the published literature (Table 2). A limitation of our study is the reliance on clinical notes to determine time to symptom relief. Documentation and follow-up time were not standardized, so our analysis may have overestimated the time to symptom relief.

Most of the side effects experienced by the patients in this study were similar to the side effects expected with imatinib or radiation monotherapy. An advantage of combining imatinib with radiation is that a lower dose of radiation (median 50 Gy) than typically used was effective. However, it is possible that the combination of imatinib and radiation could lead to increased toxicity. For example, avascular necrosis of the humeral head is a rare complication of radiation therapy [20], and imatinib may also be associated with the development of avascular necrosis [21]. As a result, it will be critical to limit the dose to the humeral head and shoulder joint when using this approach to treat axillary desmoid tumors in the future.

In summary, concurrent imatinib and radiation therapy appears to be effective at preventing further growth and relieving symptoms associated with locally invasive desmoid tumors, but future studies will be necessary to more thoroughly assess the risk and efficacy of this combination. For carefully selected patients with unresectable desmoid tumors, imatinib and radiation therapy may be a reasonable therapeutic option.

## Figures and Tables

**Table 1:** Summary of patient characteristics and response to treatment.

Age	Sex	Site	Symptoms	Radiation dose	Radiation toxicity	Imatinib toxicity	Symptom relief (days)	Follow-up (months)	Change in volume	RECIST^*∗*^
52	M	R back	Pain	50 Gy in 25 fx	Grade 1 fatigue	Grade 2 nausea	7	12	−93%	PR
70	F	L axilla	Pain, weakness, and numbness	54 Gy in 27 fx	Grade 2 fatigue, Grade 1 dermatitis, L breast edema	Grade 2 fatigue, Grade 2 nausea	360	18	−69%	SD
47	F	L axilla	Pain and numbness	50.4 Gy in 28 fx	Grade 2 dermatitis, AVN of L humeral head	Grade 1 nausea	136	3	−8%	SD
63	M	R neck and upper back	Pain	50 Gy in 25 fx	Grade 2 dermatitis	Grade 2 nausea, Grade 2 dysgeusia	42	4	−28%	SD

M = male, F = female, R = right, L = left, and fx = fractions. ^*∗*^Based on change in largest diameter, PR = partial response, SD = stable disease, and AVN = avascular necrosis.

**Table 2:** Selected previous studies of desmoid tumors treated with imatinib or radiation therapy alone.

Manuscript	Toxicity	Local control

*Imatinib alone*		
Heinrich et al. 2006 [8]	≥50% grade 3	36.8% at 1 year
Chugh et al. 2010 [7]	≥9.8% grade 3/4	66% at 1 year
Penel et al. 2011 [9]	45% grade 3	67% at 1 year

*Radiation alone*		
Nuyttens et al. 2000 [10]	22.8% total	78% at 6 years
Guadagnolo et al. 2008 [11]	10.5% moderate, 4.3% severe	68% at 10 years
Keus et al. 2013 [19]	4.5% grade 3	81.5% at 3 years
